# Matching Pursuit with Asymmetric Functions for Signal Decomposition and Parameterization

**DOI:** 10.1371/journal.pone.0131007

**Published:** 2015-06-26

**Authors:** Tomasz Spustek, Wiesław Wiktor Jedrzejczak, Katarzyna Joanna Blinowska

**Affiliations:** 1 Department of Biomedical Physics, Warsaw University, Warszawa, Poland; 2 Institute of Physiology and Pathology of Hearing, Warszawa, Poland; 3 Institute of Biocybernetics and Biomedical Engineering of Polish Academy of Sciences, Warszawa, Poland; Leibniz Institute for Age Research, GERMANY

## Abstract

The method of adaptive approximations by Matching Pursuit makes it possible to decompose signals into basic components (called atoms). The approach relies on fitting, in an iterative way, functions from a large predefined set (called *dictionary*) to an analyzed signal. Usually, symmetric functions coming from the Gabor family (sine modulated Gaussian) are used. However Gabor functions may not be optimal in describing waveforms present in physiological and medical signals. Many biomedical signals contain asymmetric components, usually with a steep rise and slower decay. For the decomposition of this kind of signal we introduce a dictionary of functions of various degrees of asymmetry – from symmetric Gabor atoms to highly asymmetric waveforms. The application of this enriched dictionary to Otoacoustic Emissions and Steady-State Visually Evoked Potentials demonstrated the advantages of the proposed method. The approach provides more sparse representation, allows for correct determination of the latencies of the components and removes the "energy leakage" effect generated by symmetric waveforms that do not sufficiently match the structures of the analyzed signal. Additionally, we introduced a time-frequency-amplitude distribution that is more adequate for representation of asymmetric atoms than the conventional time-frequency-energy distribution.

## Introduction

The time evolutions of many physical phenomena are characterized by a fast increase and slower decay. Quite often the stimulus causes a steep rise in response and then a gradual decrease. This kind of behavior is observed for some biomedical signals of non-stationary character. For the analysis of non-stationary signals many time-frequency (t-f) methods have been proposed: windowed short-time Fourier transform, Wigner-Ville transform, wavelets and adaptive approximations by Matching Pursuit (MP) [1]. Comparison of the above mentioned methods of t-f analysis shows that the method, which supplies the highest t-f resolution is MP [2,3]. The MP method decomposes the signal into waveforms (atoms) taken from a very large and redundant dictionary of functions of well-defined t-f properties. The resolution of MP is close to the limit set by the indefinity principle [4]. MP has been extensively used for the analysis of brain signals [5, 6, 7, 8, 9] and Otoacoustic Emissions (OAE) [3, 10, 11]. Apart from high t-f resolution MP has another advantages such as providing parametric description of signals, which enables variables characterizing the signal’s components to be extracted, namely frequency, amplitude, latency, time span and phase.

In MP algorithm introduced in [4], a dictionary of Gabor functions was used. Indeed, Gabor functions provide the highest t-f resolution, however some signals contain highly asymmetric components, which are ill described by Gabor functions. The problem is especially important when estimation of latency of the response is of interest.

The aim of this work is to demonstrate the usefulness of the MP method with an Enriched Dictionary (ED) i.e. dictionary which besides the Gabor functions contains functions of varying asymmetry. Additionally we propose representation of signals in t-f space by means of the amplitude maps. This kind of representation eliminates cross-terms and is more intuitive than conventional energy distribution.

Here we describe the method of adaptive approximation and then introduce an enriched dictionary containing asymmetric functions. Next, the properties of different t-f representations are investigated. Then the effects of an application of an enriched dictionary are exemplified both on simulated and on biomedical time series, in this case OAEs and Steady-State Visual Evoked Potentials (SSVEP). In a final discussion, special properties of the enriched dictionary, such as flexibility and sparseness, are set out.

## Materials and Methods

The study was approved by the Bioethical Committee of the Istitute of Physiology and Pathology of Hearing. All subjects in the study declared an absence of neurological or mental illnesses, and were screened against photosensitive epilepsy by the standard clinical EEG test. Informed, written consent was obtained from all of the subjects. No animal research was conducted.

### Signals

All of the signals processed in this study are available at the following web adress: http://zfb.fuw.edu.pl/data/MPwithAsymmetricFunctionsStudy.rar.

An OAE is a weak signal generated by the inner ear following acoustic stimulus and also sometimes spontaneously. It can be observed by means of a sensitive microphone placed in the ear canal. Studies of OAE are essential for gaining a better understanding of the mechanisms of hearing and are also important for the diagnosis of hearing impairment. They have been particularly useful in detecting hearing deficits in neonates, children and adults as a non-invasive test, not requiring the cooperation of the patient. One of the most common ways to measure OAE is by applying a short broadband stimulus (click) and recording the signals that follow. Signals generated in this way are called Transient Evoked Otoacoustic Emissions (TEOAE). The response of the ear after the broadband stimulus lasts about 20 ms and is characterized by spectra that consist of multiple peaks, distributed between 0.5 and 5 kHz. Because of the complex structure of TEOAEs, several t-f methods have been applied in their analysis, including short-time Fourier transform [12], methods based on the Wigner-Ville transform [13] or Choi-Williams transform [14], wavelet transform [15, 16], minimum variance spectral estimation [17], and adaptive approximation [3]. The last method provides the highest t-f resolution.

There is also a class of responses called Synchronized Spontaneous Otoacoustic Emissions (SSOAEs), which are generated after an acoustic stimulus, but last much longer than typical evoked components. These components are not well described by symmetric Gabor functions [10]. This observation has encouraged us to introduce a dictionary of asymmetric functions for OAE decomposition [18]. The decaying part of the asymmetric function used in [18] had the shape of an exponentially damped sinusoid. Herein we applied a more general formula providing greater flexibility.

SSVEPs are signals comprising electrical brain responses induced by flickering visual stimuli. When the retina is excited by an oscillating visual stimulus ranging from 4 Hz to 80 Hz, the brain generates electrical activity at the frequency of the visual stimulus plus harmonics. SSVEPs are periodic with a stationary distinct spectrum and are characterized by a good signal-to-noise ratio and relative immunity to artifacts. SSVEPs provide means for characterizing preferred frequencies of neocortical dynamic processes. They are used in studies concerning cognition (visual attention, working memory, brain rhythms, binocular rivalry) and in clinical neuroscience (aging, neurodegenerative disorders, ophthalmic pathologies, schizophrenia, depression, autism, migraine, anxiety, epilepsy) [19]. SSVEP also found application in the design of Brain-Computer Interfaces (BCI), e.g. [20]. Despite 40 years of investigation, the mechanisms underlying SSVEP are poorly understood, hence the need for advanced signal processing methods that are able to quantitatively describe these signals.

SSVEP are best observed in the t-f domain. Different t-f methods have been applied to the signal: chirp analysis [21], continuous complex Morlet wavelets [19] and the “bump” model [22]. However, none of these methods provided adequate t-f resolution. In particular wavelets–the most popular method of t-f analysis–give poor time resolution at low frequencies and poor frequency resolution at higher frequencies.

### Experimental procedures

OAEs were measured in 41 subjects (82 ears; 41 right, 41 left; age: 22–35 years). All subjects were laryngologically healthy and had no otoscopic ear abnormalities. Impedance audiometry gave normal type-A tympanograms and normal acoustic reflexes. Hearing thresholds were better than 20 dB HL for all test frequencies (0.25, 0.5, 1, 2, 3, 4, 6, 8 kHz). Testing was conducted using the ILO-292 apparatus (Otodynamics, UK, software version 5.6). Standard click stimuli were used to evoke a total of 260 OAE responses, which were averaged before further analysis. Stimuli were elicited at approximately 80 dB pSPL level. The inter-stimulus interval was 20 ms. An early part of the response (0–2.5 ms) was windowed automatically by the system to minimize stimulus artifacts. The sampling frequency was 25600 Hz. All recordings were performed at the default settings provided by the manufacturer.

SSVEP signals were recorded from one adult healthy person by means of a TMSI-porti 7 EEG amplifier with *openBCI* and *Svarog* software. This software is available under terms of the GPL license from http://git.braintech.pl and http://braintech.pl/svarog. 19 Ag/AgCl electrodes referenced to linked ears were placed on the scalp according to the 10–20 system were used. The reference was linked ears. The stimulation was provided by a checkerboard flickering at frequency 15 Hz. EEG. Sampling frequency was 1024Hz. The 20-second-long SSVEP records preceded by 5 seconds of resting EEG were averaged over 50 trials.

### Matching Pursuit

The method of adaptive approximation by MP introduced by Mallat and Zhang [4] relies on the decomposition of the signal into an assembly of functions from a very large and redundant dictionary. A dictionary of basic waveforms can be generated e.g. by scaling, translating and, unlike in wavelet transform, by modulating the window function *g*(*t*):

$$g_{I}\left(t\right)=\frac{1}{\sqrt{s}}g\left(\frac{t-u}{s}\right)e^{i\xi t}$$ (1)

where *s*>0 is the scale, *ξ* the frequency modulation, and *u* the translation.

Index *I* = (*ξ*, *s*, *u*) describes the set of parameters. The window function *g*(*t*) is usually even and its energy in the time domain is mostly concentrated around *u* with a variance proportional to *s*. In the frequency domain the energy is mostly concentrated around *ξ* with a spread proportional to 1/*s*.

Finding an optimal approximation of a signal by selecting functions from such a large family is an NP-hard (computationally intractable) problem [23]. Therefore a suboptimal iterative procedure is applied. In the first step of the procedure the vector which gives the largest inner product with the signal *f*(*t*) is chosen:

$$f=\langle f,g_{I_{0}}\rangle +R^{l}f.$$ (2)

Then the residual vector *R* ^*I*^ *f* is decomposed in a similar way. An iterative procedure is repeated on the subsequent obtained residues:

$$f=\langle R^{n}f,g_{I_{n}}\rangle g_{I_{n}}+R^{n+1}f,$$ (3)

where $\left\langle R^{n}f,g_{I_{n}}\right\rangle$ is the amplitude of the winning atom in the *n*-th iteration. In the procedure the signal *f* is decomposed into a sum of waveforms chosen to optimally match the signal’s residues:

$$f=\sum_{n=0}^{m}\langle R^{n}f,g_{I_{n}}\rangle +R^{m+1}f\;.$$ (4)

The point at which the iterations should be stopped, or, as an equivalent, the number of waveforms in expansion (4), can be chosen individually for each signal based on mathematical criteria or set arbitrarily, e.g. as a percentage of energy accounted for.

In the MP procedure usually functions from the Gabor family are usually used:

$$g\left(t,\mu ,\sigma ,\omega \right)=Ne^{\frac{-{\left(t-\mu \right)}^{2}}{2\sigma^{2}}}e^{i\omega t}$$ (5)

where: *N* is the normalization constant, *σ* the scale, *µ* the position in time and *ω* the circular frequency.

The method is very robust with respect to noise. The addition of noise with variance twice bigger than the variance of the signal does not critically influence the t-f positions of waveforms corresponding to simulated structures [5].

### Matching Pursuit with enriched dictionary

In order to account for the presence of asymmetric waveforms in signals, a dictionary consisting of two-sided functions is proposed. These functions are each composed of two parts: the ascending part is based on a Gabor function, and the descending part on a *atan*, which makes possible to account for the large range of decaying characteristics. Such a waveform is described by the formula:

$$\Lambda \left(N,\alpha ,\beta ,\sigma ,\omega ,\mu \right)=N\;\exp\left(\frac{-\frac{{\left(t-\mu \right)}^{2}}{2\sigma }}{1+\alpha \left(t-\mu \right)\frac{\left(\mathrm{atan}\left(\beta \left(t-\mu \right)\right)+\frac{\pi }{2}\right)}{\pi }}\right)e^{i\omega t}$$ (6)

where:

*N*–normalization factor,

β–constant (must be high enough to encompass the whole range of atan arguments from almost-∞ to +∞) we used β = 10^16^,

μ–position in time,

ω–circular frequency,

α–the descending slope of the function,

*σ*–scale (time span).

Parameters μ, α, σ, ω are fitted by the MP algorithm.

The function obtained in this way is continuous up to the first order derivative. The waveforms described by the above formula could have different rise and fall times for the same frequency. The envelope of such an atom is:

$$\Omega \left(N,\alpha ,\beta ,\sigma ,\mu \right)=N\;\exp\left(\frac{-\frac{{\left(t-\mu \right)}^{2}}{2\sigma }}{1+\alpha \left(t-\mu \right)\frac{\left(\mathrm{atan}\left(\beta \left(t-\mu \right)\right)+\frac{\pi }{2}\right)}{\pi }}\right)$$ (7)

**Fig 1:**
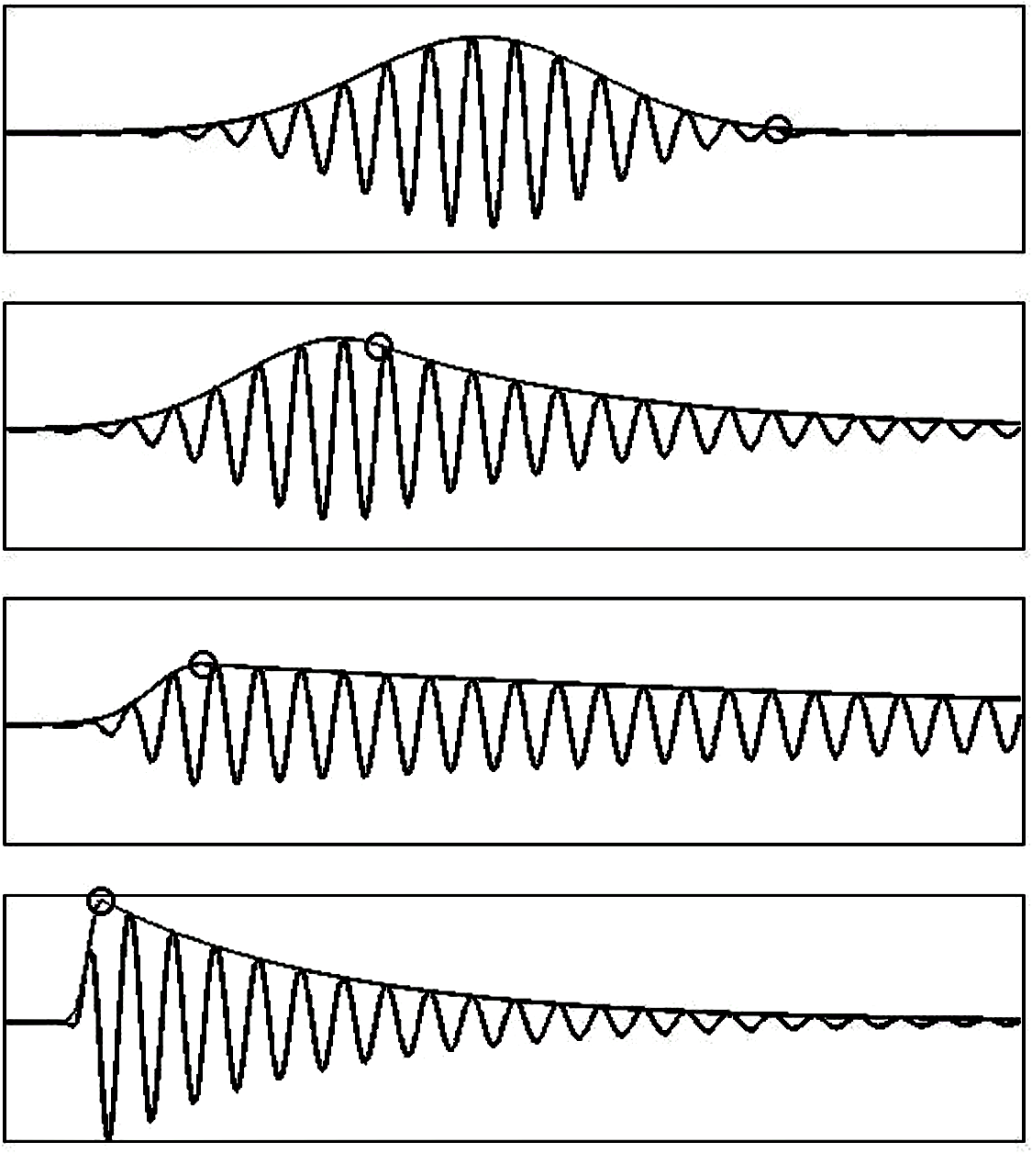
Examples of functions with different asymmetry used in the enriched dictionary.

The dictionary of functions used here consists of asymmetric functions described by Eq 6 and symmetric Gabor functions (which can be considered as a special case of the function in Eq 6). Examples of functions described by Eq 6 are shown in Fig 1. They include functions of different shapes, from almost symmetric to highly asymmetric. The ED used in our approach is larger than the standard Gabor Dictionary (GD) and its size depends on the number of asymmetries present in the signal components. Here we have used a dictionary of Gabor functions consisting of 10^7^ atoms. The ED was about 13 times bigger.

### The representation of signals in the time-frequency space

The results of MP decomposition can be visualized in the t-f plane by adding the Wigner distributions of each of the selected waveforms. The Wigner distribution is defined by:

$$W\left[f,h\right]\left(t,\omega \right)=\frac{1}{2\pi }\int_{-\infty }^{+\infty }f\left(t+\frac{\tau }{2}\right)h\left(t-\frac{\tau }{2}\right)e^{-i\omega t}d\tau$$ (8)

The t-f distribution for the decomposed signal is given by the formula:

$$W\left[f,f\right]\left(t,\omega \right)=\sum_{n=0}^{\infty }{\left|\langle R^{n}f,g_{I_{n}}\rangle \right|}^{2}W\left[g_{I_{n}},g_{I_{n}}\right]\left(t,\omega \right)+\sum_{n=0}^{\infty }\sum_{m=0,m\neq n}^{\infty }\langle R^{n}f,g_{I_{n}}\rangle \bar{\left\langle R^{m}f,g_{I_{m}}\right\rangle }W\left[g_{I_{n}},g_{I_{m}}\right]\left(t,\omega \right)$$ (9)

where the double sum in Eq 9, containing cross distributions of different waveforms, corresponds to the cross terms generally present in the Wigner distribution. One usually tries to remove these terms in order to obtain a clear picture of the energy distribution in the t-f plane. Removing these terms from Eq 9 in case of Gabor functions is straightforward–only the first sum is kept. Then the energy density in the t-f plane of the signal’s representation obtained by means of MP is given by the expression *Ef*(*t*,*ω*):

$$Ef\left(t,\omega \right)=\sum_{n=0}^{\infty }{\left|\langle R^{n}f,g_{I_{n}}\rangle \right|}^{2}W\left[g_{I_{n}},g_{I_{n}}\right]\left(t,\omega \right)$$ (10)

The distribution conserves the signal energy in the t-f space. This type of distribution was applied in most of the studies that used MP with dictionaries consisting only of symmetric Gabor waveforms e.g. [3, 5, 6, 8, 10, 11].

**Fig 2:**
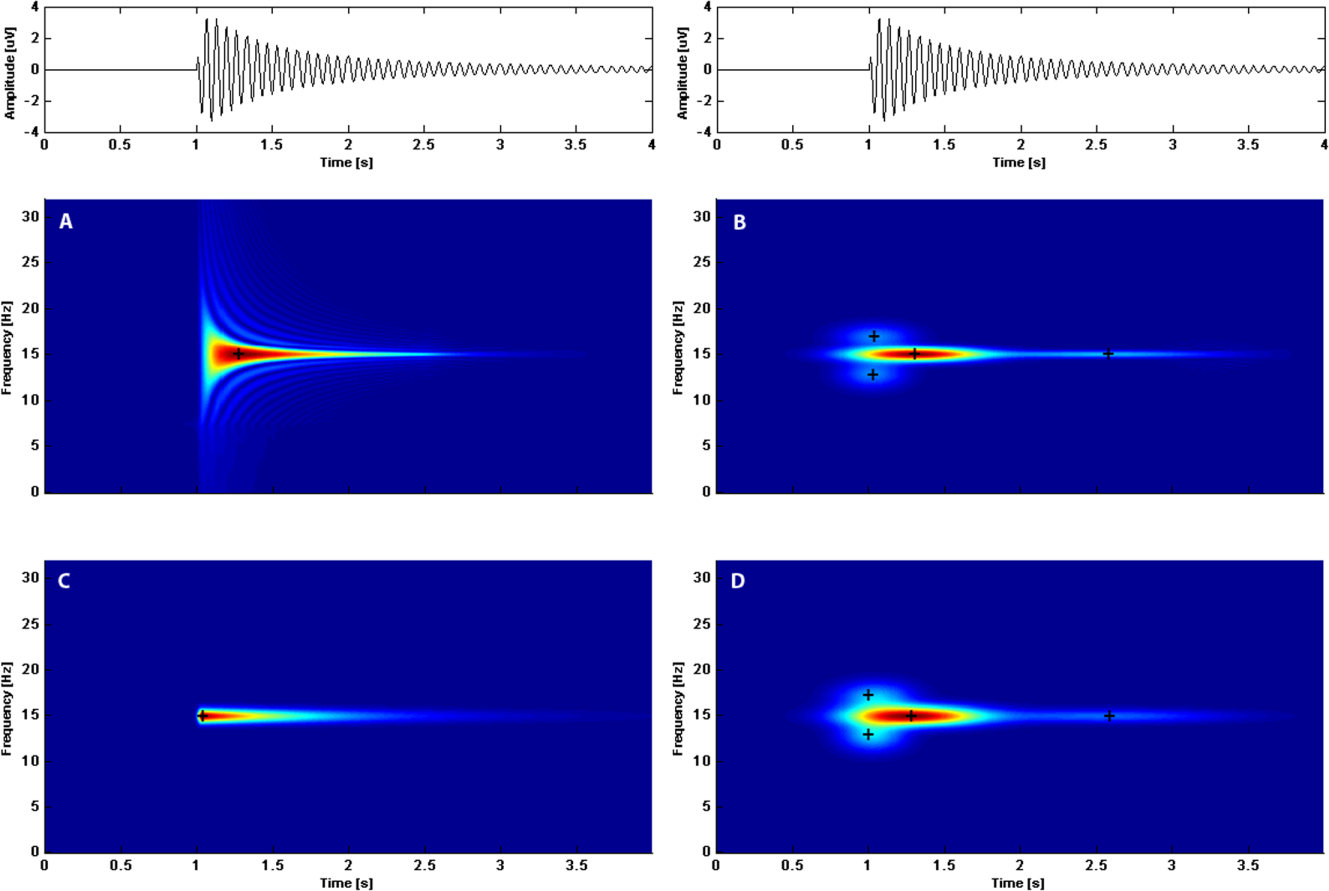
At the very top: simulated signal. Below: time-frequency-energy representations of the simulated signal obtained by: A—asymmetric dictionary and B—Gabor dictionary; time-frequency-amplitude representations obtained by: C—asymmetric dictionary and D—Gabor dictionary. Crosses mark the centers of the atoms.

However, the Wigner distribution is not an optimal choice in the case of asymmetric dictionaries. This can be illustrated with the simulation presented in Fig 2. The maximum of energy in the Wigner distribution for the dictionary of Gabor functions is shifted in relation to the maximum of of instataneous amplitude of the signal. Moreover, it is easy to see that expantion in Gabor dictionary needs 4 atoms to represent the function. In the case of ED, with asymmetric functions, only one atom is sufficient to represent energy of the function in t-f space. However, the squaring procedure inherent in the Wigner distribution creates cross-terms or “ghost-like” structures.

The inconvenience of the lack of correspondence between maximum of energy and maximum of amplitude in the case of asymmetric structures may be reduced by applying the Rihaczek distribution [24]. The Rihaczek distribution is a complex Hilbert space inner product between the time series and its infinitesimal stochastic Fourier generator. In Rihaczek distribution the maximum of energy coincides with the maximum of amplitude, but the distribution is not free from interference terms [25].

The advantage of the Rihaczek distribution in regard to the position of the energy maximum may be achieved in an easier and more intuitive way while also eliminating the “ghost structures” and interference terms. Namely, representation may be constructed as a distribution of amplitude in the time-frequency plane. The idea is based on calculation of outer product between modulus of Fourier transform of an atom and its envelope *P(t)*. In this way, we get the amplitude representation of an atom in t-f space *A(ω*,*t)*, expressed as follows:

$$A\left(t,\omega \right)=Z^{T}\left(\omega \right)\;P\left(t\right)$$

$$Z^{T}\left(\omega \right)=\left|\frac{FT\left(\Lambda \right)}{\max\left(FT\left(\Lambda \right)\right)}\right|$$ (11)

$$P\left(t\right)=\langle R^{n}f,\Lambda_{I_{n}}\rangle \;\Omega \left(N,\alpha ,\beta ,\sigma ,\mu \right)$$

where $\Lambda_{I_{n}}$ is the winning atom (function chosen from dictionary) and $\left\langle R^{n}f,\Lambda_{I_{n}}\right\rangle$ is winning atom’s amplitude. *Z* ^*T*^ *(ω)* is the Fourier transform normalized such that its maximum is equal to 1 and *P(t)* is the atom’s envelope. The time-amplitude profile of the amplitude map of a single atom gives the atom envelope. The t-f representation of the decomposed signal is a sum of the distributions given by Eq 11.

In the case of an amplitude map constructed as a sum of the distributions given by Eq 11 the cross-terms are eliminated. The latency of a maximum of a single atom in the amplitude map is the same as of the maximum of amplitude of the atom in the time domain. The amplitude representation, especially in the case of biomedical signals, is more intuitive and more compatible with the other methods used to evaluate signals, than the energy representation.

As a result of this study we developed a plug-in to the EEGLAB Matlab Toolbox available at: http://git.nimitz.pl/mp-eeglab-plugin.git (maintained git repository) or at: http://zfb.fuw.edu.pl/data/mp-eeglab-plugin.rar (static package).

## Results

### Simulations

**Fig 3:**
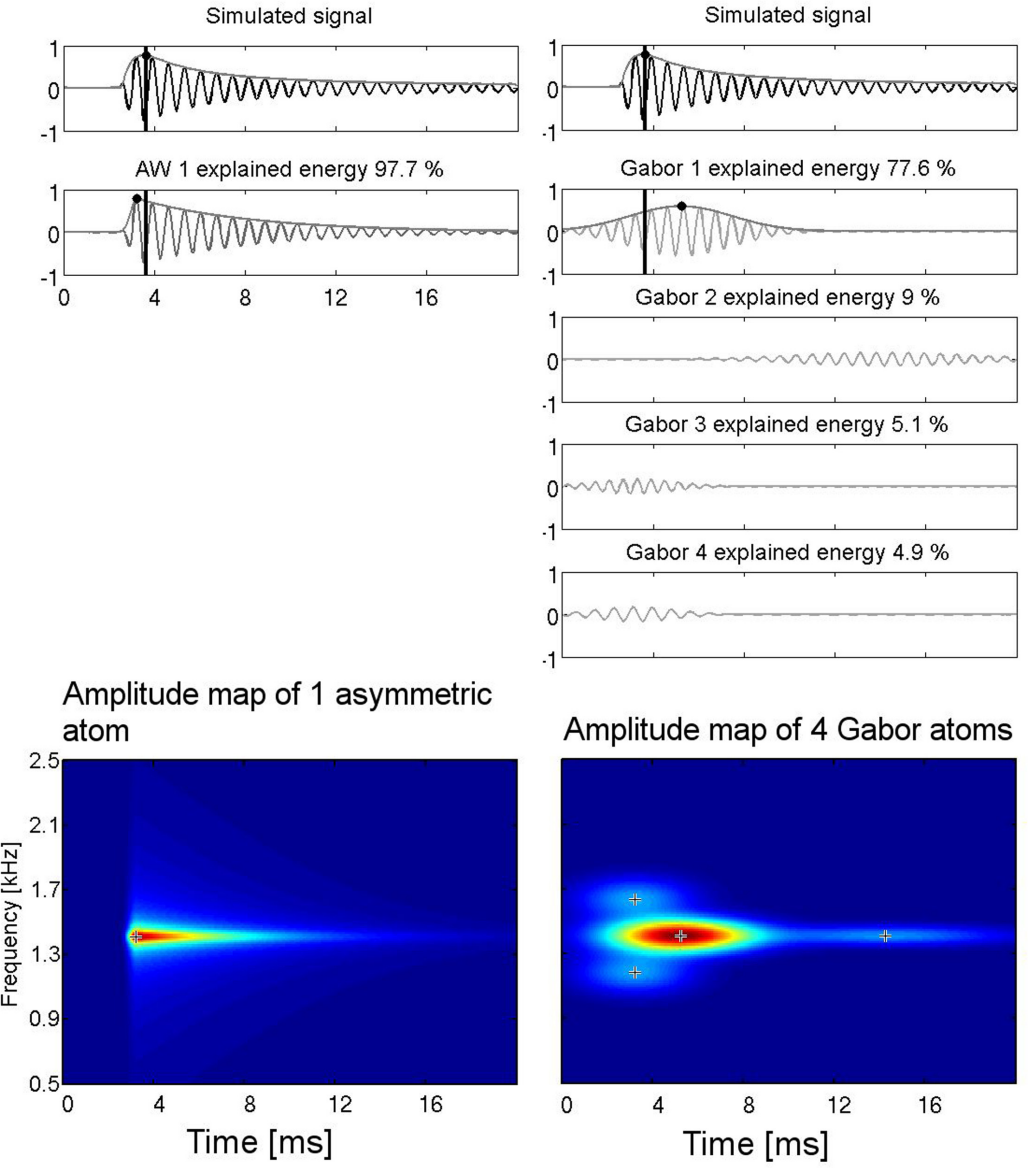
The decomposition of the function shown in the upper panels: on the left the representation by means of the enriched dictionary, on the right by means of the Gabor dictionary. In the case of Gabor dictionary, four functions are needed to account for 97.7% of energy, in case of the enriched dictionary one function alone is sufficient. Panels at the bottom: amplitude representation for enriched dictionary (left) and Gabor dictionary (right). Crosses mark the centers of the atoms.

In Fig 3 the decomposition of the simulated signal into components is shown. The simulated signal was asymmetric, but it was not constructed from functions contained in the applied dictionary. In fact, it was given by the formula:

$$f\left(t\right)=\frac{t}{10^{-6}+t^{2}}\cos\left(\omega t\right)$$ (12)

We can observe that one atom of the ED accounted for 97.7% percent of the signal energy. In the case of the GD four atoms were required to account for the same percentage of energy. The t-f-amplitude maps are shown below the decompositions. The advantages of the ED combined with t-f-amplitude map can be easily seen.

**Fig 4:**
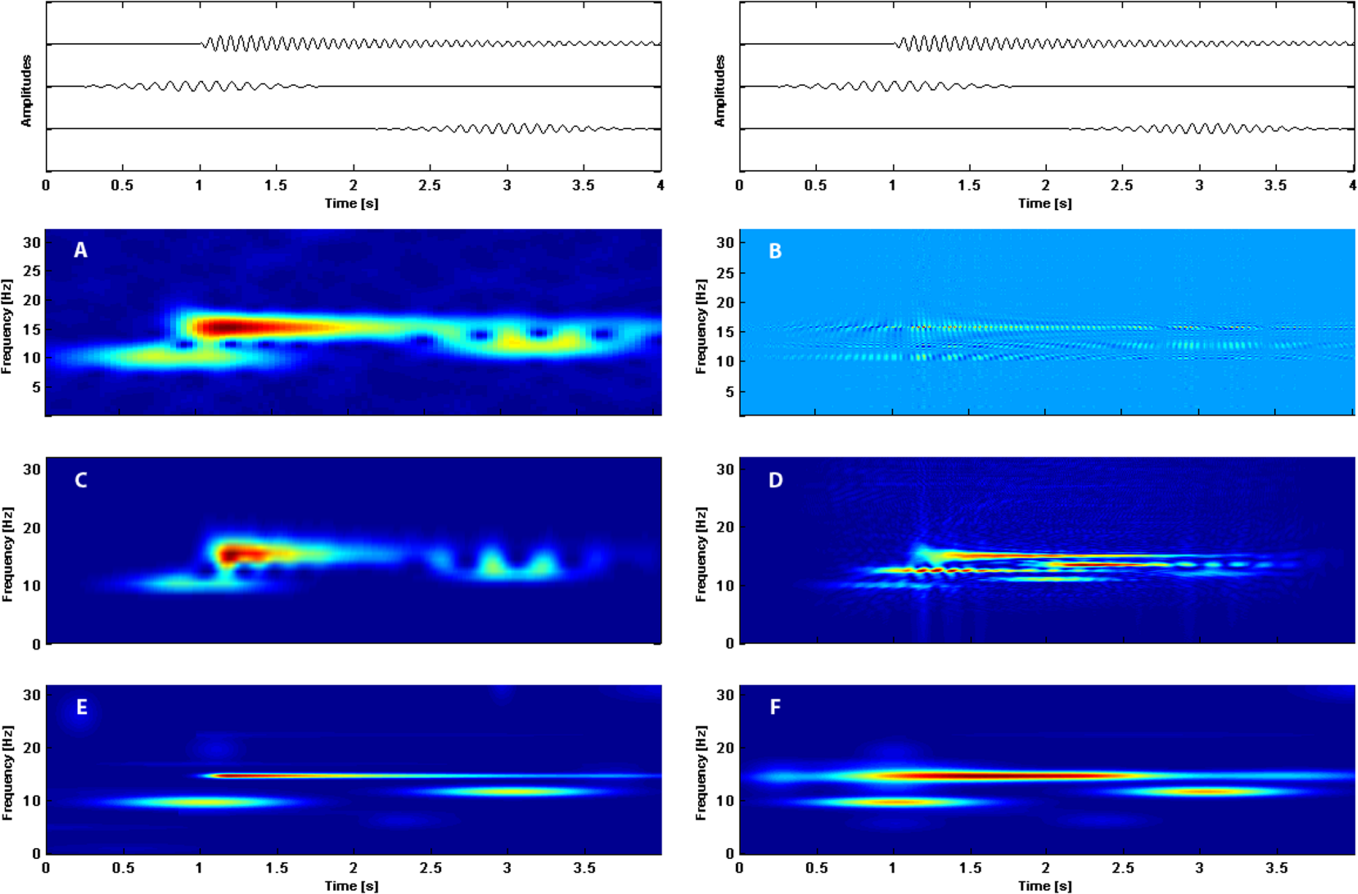
Time-frequency distributions obtained by: A—windowed Fourier transform (spectrogram), B—Rihaczek transform, C—Morlet wavelets, D—Wigner de Ville transform, E—MP with the enriched dictionary, F—MP with the Gabor dictionary. Components of simulated signal consisting of asymmetric waveform of frequency 15 Hz and two spindles of frequencies 12 Hz and 10 Hz are shown at the very top of the picture. On the horizontal axis time, on the vertical axis frequency in Hz. The colors represent: for four upper panels energy and for two lowest panels amplitude (red the strongest, dark blue the weakest).

Comparison of t-f distributions obtained by different methods–spectrogram, Rihaczek distribution, Morlet wavelets, Wigner de Wille transform, MP including enriched dictionary and MP with Gabor dictionary are shown in Fig 4. The simulated signal consisted of an asymmetric long-lasting frequency component of 15 Hz similar to SSVEP and two frequency components of 12 Hz and 10 Hz of a spindle-like shape similar to alpha waves. White noise with the amplitude equal 10% of the synthetic signal was added. In all representations we can distinguish three components but with the differing resolution. The t-f resolution in the case of the spectrogram is not very good and representation of the 12 Hz component is corrupted by the 15 Hz wave. In the case of Rihaczek distribution and Wigner de Ville transform, strong interference terms occur. Additionally the latencies and time spans are not correctly reproduced. MP with the GD for alpha waves gives very good results, but for asymmetric component of 15 Hz the pre-echo effect is visible, and the latency of the component is not quite correct. For MP with the ED the representation is more than satisfactory. The MP based t-f distributions display the amplitude of the signals, not the energy therefore they are free from interference terms (for the GD they are absent anyway, but they appear for asymmetric functions). Concluding, we may say that MP with the dictionary including asymmetric functions and amplitude-time-frequency distribution is an optimal method of signal representation in t-f space.

### Application to OAE

**Fig 5:**
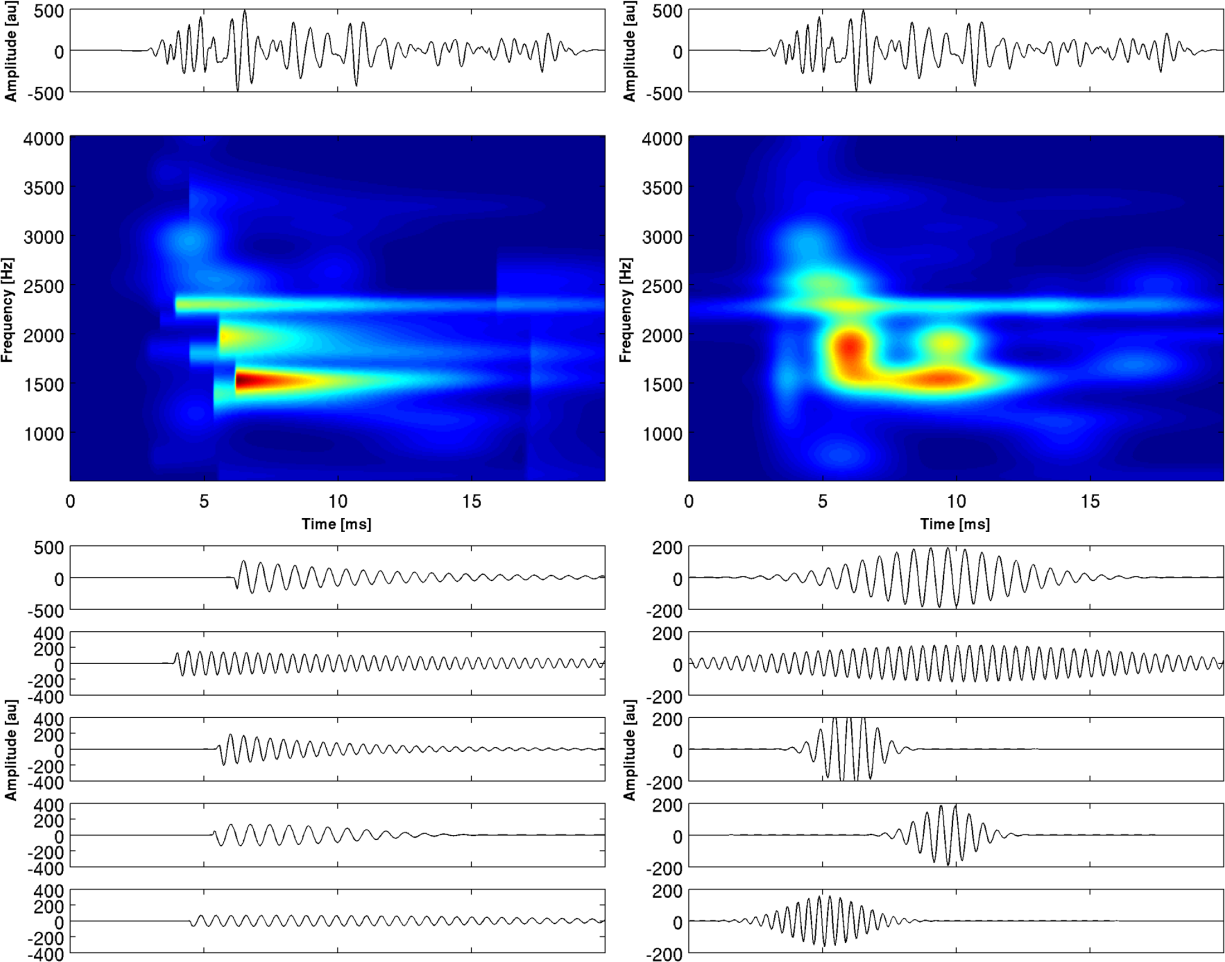
The decomposition of the TOAE signal shown at the very top; obtained by the enriched dictionary (on the left) and by Gabor dictionary (on the right). Below the time-frequency-amplitude maps, at the bottom the first five (strongest) atoms of the decomposition. The maxima of amplitudes of the first five atoms are marked by crosses.

OAEs are the time series in which the application of the ED appears to be indispensable An example of the decomposition of TEOAE by means of enriched and Gabor dictionaries is shown in Fig 5. Inspecting the t-f map we can observe, in the case of GD, "energy leakage"- energy preceding the stimulus. The effect is especially clearly visible in the second strongest frequency component of 2.30 kHz. Fig 5 shows that the GD does not represent well the long-lasting components of a steep rise. They are approximated by more than one atom.

The MP decomposition is performed in an iterative way, first fitting to the signal the atom accounting for the most of the energy and then repeating the procedure on the following residues. In the lower part of Fig 5, five atoms of the decomposition are displayed in order of decaying energy. In Table 1 and Table 2, parameters describing five strongest atoms are shown. In the case of ED the first 5 atoms correspond to five asymmetric frequency components: 1.56 kHz, 2.30 kHz, 2.02 kHz, 1.38 kHz and 1.78 kHz. The first two atoms of the GD have similar frequencies to the ED: 1.56 kHz and 2.30 kHz, but their latencies are longer (Table 1 and Table 2). Atoms 3 and 4 of the GD have close frequencies (1.86 kHz and 1.90 kHz) similar to the frequency of the third atom of ED, so it seems that this is the same component of the TEOAE signal, but the GD was unable to fit one atom to it. In the GD t-f map, we can observe that the long structure at 2.3 kHz consists of several atoms. Inspecting the t-f plot at Fig 5, we can also see also that there is a small structure of roughly 1.6 kHz and latency around 9 ms below the third atom (1.86 kHz). This structure, together with the strongest atom (at 1.56 kHz) of GD decomposition, probably accounts for the same long-lasting component described in the ED as one waveform of 1.56 kHz frequency. These examples show that the representation of TEOAE provided by the ED is sparser. One atom of the ED being described in some cases by two or more atoms of the GD.

**Table 1:** Parameters of TOAE structures provided by enriched dictionary.

#	Amplitude [au]	Width [s]	Latency [s]	Frequency [Hz]
1	279.37	0.0038	0.0065	1564.61
2	163.52	0.0099	0.0044	2304.21
3	207.78	0.0032	0.0060	2017.00
4	135.52	0.0057	0.0060	1379.30
5	69.56	0.0133	0.0060	1777.52
6	84.96	0.0040	0.0057	2549.01

**Table 2:** Parameters of TOAE structures provided by standard Gabor dictionary.

#	Amplitude [au]	Width [s]	Latency [s]	Frequency [Hz]
1	190.37	0.0064	0.0097	1565.19
2	116.68	0.0145	0.0113	2299.00
3	288.69	0.0020	0.0060	1863.93
4	194.22	0.0023	0.0095	1899.29
5	160.79	0.0032	0.0053	2516.50
6	96.72	0.0049	0.0089	1387.25

An important parameter for TEOAE analysis is the latency of components. One can see in Fig 5 that the latencies of long-lasting components detected by the GD are usually longer in case of asymmetric components. When more than one atom is needed to describe a given waveform the latency of the component is ill defined. The exact identification of TEOAE latencies is important in clinical diagnosis [10].

### Application to SSVEP

**Fig 6:**
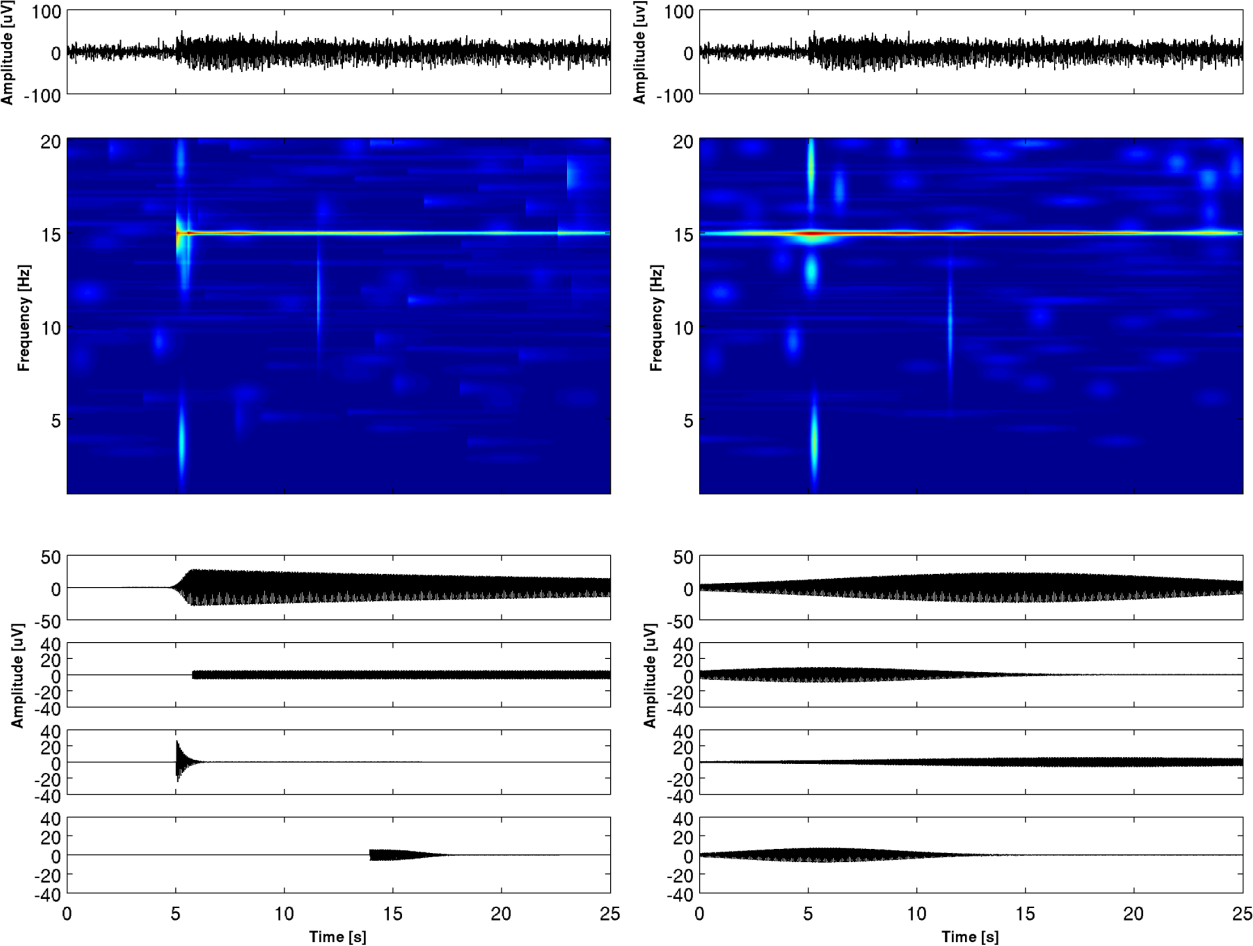
The decomposition of the SSVEP signal recorded at electrode O2 (shown at the very top) obtained by the enriched dictionary (on the left) and by Gabor dictionary (on the right). Below: time-frequency-amplitude maps, at the bottom the first five atoms of the decomposition. In Table 3, parameters (frequency, amplitude, time occurrence and time span) describing the first five atoms are shown. In the time-frequency map harmonics at 30 Hz is not shown in order to make the figure more compact.

For SSVEP analysis we applied the MP method with the dictionary encompassing asymmetric waveforms and for comparison the dictionary with symmetric atoms only. In Fig 6 the time-frequency-amplitude maps and the five strongest atoms obtained by decomposition of the SSVEP registered at electrode O2 are shown for the enriched and Gabor dictionaries. The t-f representations reveal the very narrow-band characteristics of SSVEP. In the case of Gabor representation we can observe the “energy leakage” effect–the energy of the signal appears before the start of the signal. The symmetric dictionary is unable to clearly show the onset of SSVEP. In the case of the ED the first and second atoms correspond to the basic frequency of 15 Hz and its harmonics. However, in the case of the GD only the third atom corresponds to the harmonics, since the second atom is needed to improve the representation of the 15 Hz component. In the case of the ED the third atom of very short duration represents the fast onset of SSVEP, and its latency of 5.05 ms corresponds very well to the start of the stimulus. The GD does not allow accurate determination of the onset of SSVEP. In Table 3 and Table 4, amplitudes, frequencies, latencies and time spans of the displayed atoms are given. It is easy to see that for both representations the frequencies of harmonics reproduce the stimulating frequency very accurately.

**Table 3:** Parameters of SSVEP structures provided by both dictionaries in O2 channel.

#	Amplitude [au]		Width [s]		Latency [s]		Frequency [Hz]	
Dictionary	E	G	E	G	E	G	E	G
1	28.54	23.31	19.70	19.47	5.94	14.29	14.98	14.98
2	5.22	9.26	19.24	11.49	9.77	5.66	29.95	14.90
3	28.60	5.95	0.26	17.53	5.05	18.41	14.85	29.95
4	5.83	7.60	2.42	8.26	14.19	5.76	19.85	15.07

(E-enriched dictionary, G-Gabor dictionary)

**Table 4:** Parameters of SSVEP structures provided by both dictionaries in P4 channel.

#	Amplitude [au]		Width [s]		Latency [s]		Frequency [Hz]	
Dictionary	E	P4	E	P4	E	P4	E	P4
1	10.52	9.17	19.14	18.92	6.43	14.77	14.98	14.98
2	8.03	6.54	12.03	13.55	6.33	12.44	29.95	29.95
3	29.10	3.34	0.30	11.81	5.31	5.51	3.17	15.06
4	15.71	29.11	0.48	0.30	5.29	5.31	13.10	3.17

(E-enriched dictionary, G-Gabor dictionary)

**Fig 7:**
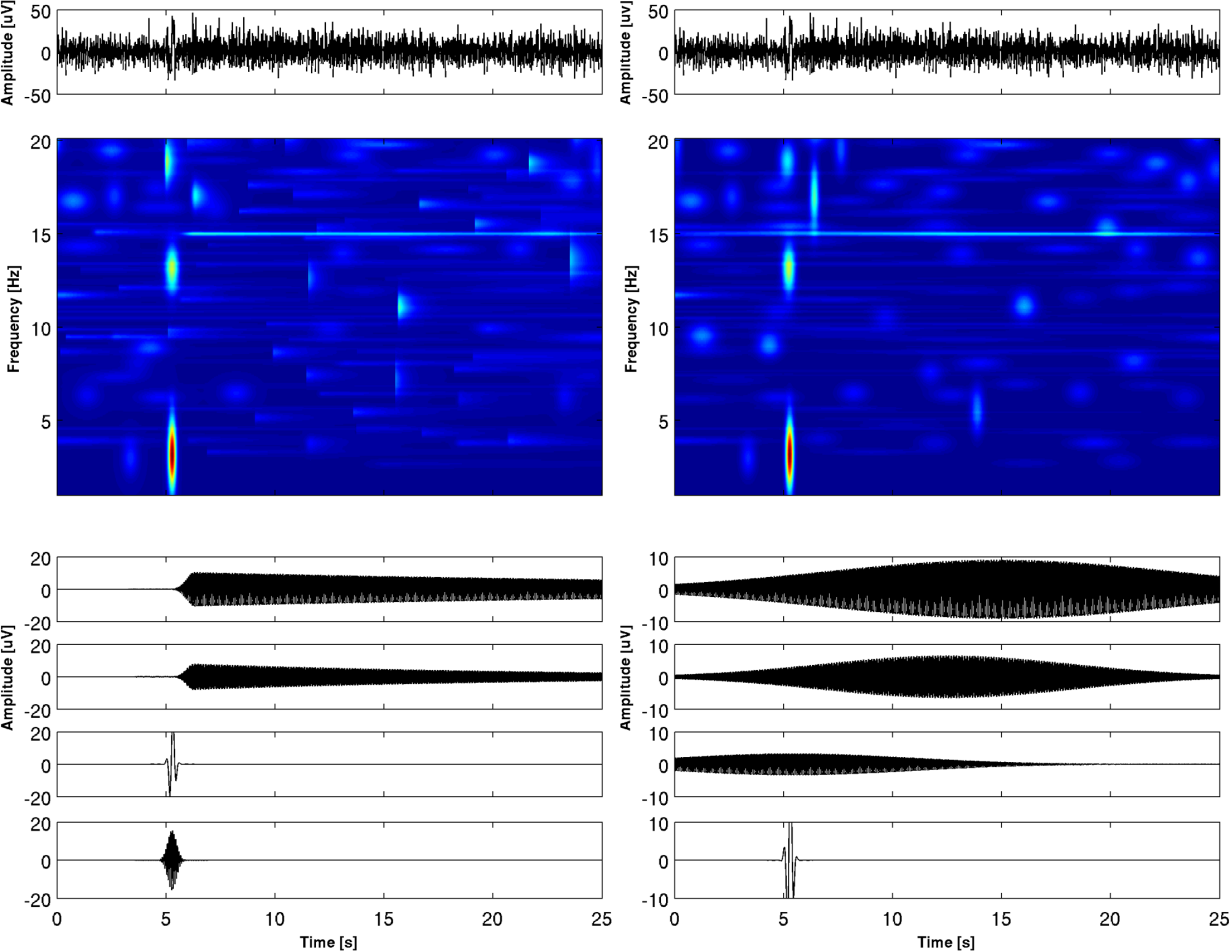
The decomposition of the SSVEP signal recorded at electrode P4 (shown at the very top) obtained by the enriched dictionary (on the left) and by Gabor dictionary (ont right). Below: time-frequency-amplitude maps, at the bottom the first five atoms of the decomposition. In Table 4, parameters (frequency, amplitude, time occurrence and time span) describing the first five atoms are shown. In the time-frequency map harmonics at 30 Hz is not shown in order to make the figure more compact.

The t-f pattern shown in Fig 6 is characteristic of the electrodes placed in the location where SSVEP is the strongest. For the electrodes further away from the place of SSVEP generation, the t-f characteristic is different. In Fig 7 the t-f map and decomposition of SSVEP into the first four atoms are shown for electrode P4. For the ED the first and second atoms correspond to the 15 Hz component and its harmonics and the third atom reveals structure similar to VEP (as its time span, 300 ms, roughly corresponds to the time span of VEP). This kind of decomposition appears for the posterior, central and frontal electrodes. For the GD this VEP-like structure is also present, but it usually occupies a lower position in the energy ranking of the atoms than the corresponding atom from the ED. It is interesting that VEP is hardly visible for the occipital electrodes, where it is masked by the SSVEP. We can see that the representation of SSVEP by means of a dictionary with asymmetric functions has a greater amount of information and may be very useful for the study of these signals.

We shall not describe in detail the features of SSVEP decomposition by MP, since it is not our aim to investigate the properties of these signals, which would require a separate study of the larger material. However, we would like to emphasize the advantages of the proposed representation. First of all, representation encompassing asymmetric waveforms is sparser. Taking into account all 19 electrodes, the average number of atoms needed to describe 95% of energy was for the GD 2.73 ± 1.28, and for the ED 1.67 ± 0.82 atoms. Secondly application of Gabor dictionary creates “energy leakage” in t-f representation, which appears before the onset of SSVEP. Symmetric waveforms are not well suited to describing the steep rise of SSVEP. The time of the appearance of SSVEP can be very well estimated by means of decomposition into asymmetric atoms, which is not the case for symmetric ones. The examples presented in Figs 6 and 7 show topographically the different t-f characteristics of SSVEP and indicate the possibility of following its evolution across the brain, which may be helpful in revealing the mechanisms of SSVEP generation.

## Discussion

The application of asymmetric waveforms for adaptive approximation of signals has already been proposed in the field of musical signals. In [26, 27], damped sinusoids were proposed as basic functions. They well describe musical tones, which have a very sharp onset, but seem to be less appropriate for other signals such as OAE and SSVEP, in which the rising part of the signal is less steep. A similar argument holds for the asymmetric functions introduced in [28], which consisted of cosine and exponential functions with fixed parameters. These kinds of functions were tailored for the representation of music and specifically for recognition of basic notes, but they are less appropriate for a more general class of signals with components of varied degree of asymmetry. The dictionary built from symmetric and asymmetric oscillatory components, namely the Epsilon-Skew-Normal dictionary for multichannel MP decomposition was proposed in [29], however its advantages for experimental signal analysis were not clearly demonstrated.

Asymmetric functions in our dictionary are very flexible. Different kinds of wave shapes may be designed by changing one parameter only. As is easy to observe by examining Figs 4–7, the introduced functions well account for structures with different degrees of asymmetry. We may say that they constitute a general framework which is useful for different kinds of signals.

The computation time of the procedure depends on the size of the dictionary, which depends on t-f resolution, which in turn is connected with a t-f grid on which the atoms are fitted. Here, we have used a high resolution, namely for OAE signals Δ*t* = 0.2 ms and Δ*f* = 12.5 Hz. In the case of SSVEP the time resolution was Δ*t* = 8 ms and the frequency resolution 0.064 Hz.

In our earlier implementation of asymmetric functions [18] the shape of the function consisted of two functions. The ascending part was described by Gabor function and the descending one by exponential function. Here, we used the analytical function *atan*, which allowed the usage of gradient descent methods in the approximation procedures. In consequence, the time of calculations decreased by a factor of 5. The time of calculations can be further reduced by the application of a dictionary with a smaller t-f resolution, since in some applications a very dense dictionary, such as we have used here, is not needed.

We have proposed another kind of t-f representation of signals, namely amplitude representation, which is free from cross-terms. A broadly applied representation of t-f distribution in terms of energy comes from electrical engineering, where power is of basic interest. Also, more traditional methods of analysis do not allow easy determination of the amplitude values of signal structures. In the case of biomedical signals amplitude is of primary interest, since it is more understandable for medical doctors and biologists, its link with the signal itself being more intuitive. It seems that in other fields of research as well, representation in terms of time-frequency-amplitude may have advantages other than the elimination of cross-terms. It was shown in [4] that the dictionary consisting of Gabor atoms provides a t-f resolution close to the theoretical limit, while in case of asymmetric functions the resolution is lower. However, in practical applications, there are advantages more important than resolution. The representation by means of asymmetric functions is more sparse than for Gabor functions. For the description of long-lasting OAE or SSVEP components more than one symmetric atom is needed. The percentage of energy that accounted for the same number of iterations performed by means of both methods varied, depending on the number of asymmetric structures. However, it was always higher for the ED, unless the number of iterations was high and was approaching the residues connected with noise.

In the case of TEOAE, the advantages of applying adaptive approximations have already been demonstrated. For example by means of MP it was first possible to identify basic components and connect them with the resonance modes of the cochlea [3]. Further improvement in TEOAE analysis was introduced by enriched dictionaries that made it possible to avoid describing one long-lasting component by several atoms and to eliminate the “pre-echo” or so-called “energy leakage” effect–the artifact connected with the occurrence of energy before the start of the actual signal.

An important aspect of the proposed method is the possibility of accurately determining of the latency of components. In the case of OAE signals, this is an important parameter for understanding the mechanisms of OAE generation, namely through comparison of the models with the experimentally estimated latency-frequency dependence. The accuracy of the estimation of this function is crucial for testing physiological models. Latency is also a parameter that is used in clinical diagnosis of hearing disorders [10, 11]. There have been attempts to identify the latencies of OAE components by means of wavelet transform [30], but the procedure did not yield the satisfactory results. Delays of components of different frequencies were found in an animal study in which OAE recorded for sweeping stimulating frequency was followed by Fourier analysis [31]. In the MP approach, latency is given directly as a parameter of the fitted function. The problem of paradoxically long latencies found in newborns was explained through the application of the MP procedure [10], which revealed the important contribution of SSOAE (i.e. long-lasting components) in the OAE of very young children. However, the correct estimation of the latencies of long-lasting components became possible only after application of a dictionary containing asymmetric functions.

The application of the ED to SSOAE made it possible to find the correspondence between the ratios of SSOAE frequencies and the musical scale [32]. In this respect, a special feature of the ED was its sparseness, which made it possible to avoid representation of one long-lasting component by means of several atoms.

Quite a few t-f analyses of SSVEP have been performed up to now. Wavelet transform was applied to SSVEP in [33, 34, 35]. In [21], adaptive chirplet transform was used. Vialatte [36] applied to SSVEP so-called “bump” analysis based on complex Morlet wavelets followed by construction of "bumps" based on half ellipsoid basis functions. However, these methods suffered from the limitation of t-f resolution inherent in the wavelet method [5, 6]. MP outperforms other methods of t-f analysis in several aspects [1, 2, 3]. From the examples shown in Figs 6 and 7 we can see that in the case of SSVEP the frequency parameter is well determined by MP with Gabor dictionary but this is not the case for latencies of components. For studies devoted to the investigation of the SSVEP generation mechanism, paradigms connected with cognition processes, or applications in clinical neuroscience, description by asymmetric functions (which better reproduces the character of SSVEP) seems indispensable.

Various theories have been put forward to explain the complexity of SSVEP [19]. These theories assume different pathways of visual stimulus propagation and different distributions of the active dipoles in the brain. The representation of SSVEP by means of basic components of well defined in t-f and described parametrically in t-f domain may help in unraveling the mechanisms underlying SSVEP. The topographical inspection of SSVEP components may elucidate the propagation of the signal and help in understanding the complexity of SSVEP.

SSVEP are used in neurophysiological investigations as frequency tags. Propagation of the SSVEP peak frequency from the primary visual areas to other brain structures is used to track the neural dynamics correlated with attention [19]. In working memory tasks, the memory load may be estimated according to the decrease of SSVEP amplitude in relation to the distance from the source [37]. In these kinds of investigations, the detailed correct description of SSVEP components and accurate determination of their frequencies seem indispensable.

SSVEP are also used for investigation of aging and psychiatric disorders such as schizophrenia and depression. SSVEP latency is delayed for schizophrenic patients [38]. Topographical differences in SSVEP were observed for patients suffering from major depression [39]. The list of SSVEP applications in neuroscience and in clinical neurology is long and it seems that the adequate representation of this signal in the t-f domain may be helpful in a wide range of clinical applications.

One of the advantages of the proposed method is the parametric description of the components in terms of their frequency, latency, time span, amplitude and degree of asymmetry. These kinds of parameters have a clear physical meaning and could be helpful in revealing the mechanisms of signal generation. Parametric description facilitates the statistical analysis of results, finding the meaningful functional dependencies between values characterizing components, and testing models.

## Conclusion

Here we have proposed a general method of decomposition of time series into basic components described by means of parameters of clear meaning. The enriched dictionary of functions allows the description of a large class of signals containing structures of broadly varying degree of asymmetry. It provides flexible and sparse representation of the time series. We have introduced the representation of signals in the time-frequency-amplitude space, which apart from eliminating spurious structures from the representation, offers a new perspective on signal representation. The presented results obtained for signals of differing origin indicate that the proposed approach may have a potentially broad range of application in different branches of biomedical research.
